# Present hedonism and future time perspectives predicting hypersexuality and problematic pornography use

**DOI:** 10.3389/fpsyt.2022.914919

**Published:** 2022-08-23

**Authors:** Mónika Koós, Gábor Orosz, Zsolt Demetrovics, Beáta Bőthe

**Affiliations:** ^1^Doctoral School of Psychology, Eötvös Loránd University, Budapest, Hungary; ^2^Institute of Psychology, Faculty of Education and Psychology, Eötvös Loránd University, Budapest, Hungary; ^3^Université d'Artois, Unité de Recherche Pluridisciplinaire Sport Santé Société, France; ^4^Centre of Excellence in Responsible Gaming, University of Gibraltar, Gibraltar, Gibraltar; ^5^Département de Psychologie, Université du Québec à Trois-Rivières, Trois-Rivières, QC, Canada; ^6^Département de Psychologie, Université de Montréal, Montreal, QC, Canada

**Keywords:** compulsive sexual behavior disorder, hypersexual behavior, problematic pornography use, time perspective, out-of-control sexual behaviors

## Abstract

The Time Perspective (TP) theory (i.e., the psychological experience regarding time) was often examined in association with different addictive behaviors, and the different TPs (i.e., Past Positive, Past Negative, Present Hedonistic, Present Fatalistic, and Future TPs) demonstrated different relationship patterns with them. However, most studies were conducted in relation to substance use-related disorders, leaving crucial knowledge gaps concerning the associations between TPs and potential behavioral addictions. The aim of the present study was to examine the associations between the five TP dimensions, hypersexuality, and problematic pornography use (PPU), considering potential gender differences. Participants from two independent samples (N_1_ = 554; N_2_ = 453) completed a self-report survey on TPs and sexual behaviors. Structural equation modeling results indicated that the Present Hedonistic TP had a positive, moderate, and the Future TP had a negative, weak association with hypersexuality in both samples. Only the Present Hedonistic TP had a significant, positive, weak-to-moderate association with PPU across the samples. The explained variances of the models were higher in case of hypersexuality (28 and 27%, respectively), than in case of PPU (1 and 14%, respectively). No significant gender differences were observed. In line with previous findings concerning other predictors of hypersexuality and PPU, the results of the present study suggest that hypersexuality and PPU may differ from each other in terms of their TP background. Yet, present hedonism, which is related to impulsivity, may play an important role in both problematic sexual behaviors, suggesting that interventions focusing on this TP might be successful in reducing hypersexuality and PPU.

## Introduction

### Hypersexuality and problematic pornography use

The prevalence of excessive and out-of-control sexual behaviors [3–10%; (1–5)] are similar to depression, anxiety or other mood disorders (6–8). Significant gender differences were observed not only in the prevalence of these behaviors (men: 5.2–18.3%, women: 1.2–7%), but also in clinical features, comorbidity, and potential psychosocial consequences (9). The body of research on the subject have increased in the past decades (3) but no consensus has been reached regarding its conceptualization (3, 10, 11). Currently, compulsive sexual behavior disorder (CSBD, i.e., uncontrollable sexual behaviors resulting in significant adverse consequences and distress) is included in the 11th edition of the *International Statistical Classification of Diseases and Related Health Problems* [ICD-11; (12)] under the impulse control disorders category.

Not only the classification of the two problematic behaviors have been debated (13–15), but their background and core diagnostic features as well. While there is a considerable amount of evidence that one of the core elements of hypersexuality is mood modification, namely using these behavioral patterns in order to cope with negative mood states such as sadness or anxiety (4, 16, 17), this diagnostic criterion was not included in the ICD-11 (12). Moreover, it appears under the impulse control disorders category, due to the repeated acts despite the harm it causes in the person's life, and the association with the uncontrollable impulses the person experiences (18), and not under the disorders due to addictive behaviors, where gaming and gambling addictions are categorized. However, some argue that classification and suggest to categorize it as a behavioral addiction because of the similar neurobiological background of hypersexuality and other, substance-related addictions (14, 19–21), and other features (e.g., unable to resist the urges/impulses, despite gaining little or no satisfaction from the behavior anymore) that are related to addictive behaviors (11).

While problematic pornography (PPU) use is listed as a possible manifestation of hypersexuality (12, 22), there is a growing number of studies (23–25) suggesting that it might show some dissimilarities with hypersexuality and might be considered as a more independent disorder, rather than a sub-type. Still, other studies indicated several similarities regarding PPU use and hypersexuality in terms of the subjective feeling of lost control over the behavior, neglecting obligations toward friends, family or workplace, impairments in the romantic relationship and other negative consequences (26). Therefore, the aim of the present study was to explore potential similarities and dissimilarities between hypersexuality and problematic pornography use by examining their associations with time perspectives, considering potential gender differences.

### Time perspectives and their associations with risk behaviors

While monitoring time is a basic function of ours, the psychological experience of this process may vary by person to person. Coding information into past, present, and future temporal frames and later using these to form expectations and make decisions can be situationally determined, but at the same time, much depend on the given individual (27). The Past Positive Time Perspective (TP) represents reminiscing in a positive, nostalgic way, while the Past Negative TP means remembering the past in a negative, unsatisfied, or even aversive way. Impulsiveness and “*carpe-diem*” mentality are the characteristics of the Present Hedonistic TP, whereas the Present Fatalistic TP describes a more helpless attitude toward the future and life in general. The last TP, the Future TP, reflects a conscientious, future goal-oriented mentality (27).

Certain categories of this experience of time might relate to different psychological or behavioral constructs. Present and future orientations tend to affect one's behavior more, while past orientations may be more likely to be associated with mood and emotions (27–29). For example, the Past Negative TP usually has negative associations with several wellbeing indicators, such as life satisfaction (30, 31) positive affect (32) and happiness (33). In contrast, the Future TP is often associated with higher levels of achievement and motivations (34–37), subjective wellbeing (38) and conscientiousness (27). The different TP orientations might have predispositional qualities regarding well- and ill-being outcomes (39), but as they might be flexible over time, and sensitive to interventions, implementing changes in the dominating temporal frames can result in better health-related outcomes (40–43).

Several studies linked risk-taking behaviors to the TP theory, specifically to the Future and Present Hedonistic TPs (28, 44–46). Furthermore, the TP theory was studied in relation to other addictive behaviors, such as smoking (47, 48), substance use (49), alcohol use (50) and problematic internet use (51–53), as addictive behaviors can be considered as prime examples of stimulating risk-taking, when the difficulties come from resisting the immediate rewards. The associations between TPs and addictive behaviors seem to be consistent throughout the aforementioned studies as risk-taking behaviors had a negative relationship with the Future TP, and a positive relationship with the Present Hedonistic TP. These associations could partly be explained by the altered cognition of individuals with problematic behaviors, the emotional regulation problems and impulsivity, which are further discussed in the next paragraphs.

Cognitive distortion and biases in the overall temporal experience of addiction has been established in the literature before (40, 54, 55). Preoccupation with the present, and an immediate pleasure, while having diminished sense of the further consequences in the future, is a characteristic of substance use disorders as well as behavioral addictions (40, 56). This association could be demonstrated in a lab environment with delay-discounting tasks, where individuals with addiction problems tend to choose a smaller, available reward instead of a larger one in the future (57). Therefore, the relationship with Future and Present Hedonistic TPs could be explained by the tendency of devaluing outcomes and focusing on the here and now.

Moreover, emotion regulation difficulties, and thus making impulsive decisions, might play a role in developing problematic behaviors over time (58, 59), and were also linked to hypersexuality (26, 60, 61) and PPU (62) directly. In a previous study, emotional dysregulation was predicted negatively by the Future TP, and positively by the more maladaptive TPs, like Past Negative and Present Fatalistic TPs (63).

To our knowledge, no previous study has previously investigated the associations between the dimensions of TP and hypersexuality and PPU. However, impulsivity is an important characteristic of out-of-control sexual behaviors (61, 64, 65), which can manifest in the lack of ability to resist the impulsive urges despite the long term negative consequences (66). Hypersexuality is often linked to the consequences of risky sexual behaviors, especially to the potential risk of sexually transmitted infections like Human Immunodeficiency Virus (67–69), financial problems (70) or physical abuse (71).

### The aim of the present study

Based on previous empirical and theoretical works (47–50), the aims of the present study were to examine the associations between the five TPs, hypersexuality, and PPU and identify similarities and dissimilarities between these two excessive sexual behaviors in terms of TPs. We hypothesized that the Present Hedonistic TP would positively (57, 72, 73), and the Future TP would negatively relate to hypersexuality and PPU (49, 74). Furthermore, the study aimed to explore possible differences between men and women regarding these associations, as previous studies reported crucial gender differences not only in the prevalence and patterns of the problematic sexual behaviors (2, 75), but regarding their background as well (23). We tested potential gender differences in an exploratory manner.

## Method

### Procedure and participants

The present study was approved by the Institutional Review Board (IRB) of the research team's university and conducted in accordance with the Declaration of Helsinki. Two separate data collections were conducted *via* several social media platforms (e.g., topic-irrelevant Facebook groups). In both cases, participants were first informed about the content and aims of the study, then, informed consent was acquired before data collection. Both surveys continued with the questionnaire assessing the time perspective theory, followed by the assessing hypersexuality, then PPU. It took ~9 min to complete the first, and 25 min to complete the second survey. No financial compensation was offered for participation.

The two samples included 554 and 453 respondents (women_1_ = 274; 49.5%; women_2_= 268; 59.2%) who were aged between 18 and 77 years (*M* = 27.36 years, *SD* = 9.39) in case of Sample 1, and between 18 and 67 years (*M* = 27.51, *SD* = 10.19) in Sample 2. Concerning the level of education, 195 and 175 participants had a degree in higher education (Sample 1: 35.2%; Sample 2: 38.9%), 257 and 254 had a high school degree (Sample 1: 46.4%; Sample 2: 56.1%), 46 and 11 had a vocational school degree (Sample 1: 8.3%; Sample 2: 2.4%) and 56 and 12 finished 8 or less classes in primary school (Sample 1: 10.1%; Sample 2: 2.6%). A total of 46.4 and 60.5% of the samples (Sample 1: 257; Sample 2: 274 participants) reported to be still in school (high school, higher education, or other programs), while 79.2 and 55.6% reported (Sample 1: 439; Sample 2: 252 participants) working full time or part time. Detailed demographic data and are available in Appendix 1.

### Measures

The Short version of the **Zimbardo Time Perspective Inventory** [ZTPI-17; (76)] includes 17 items from the original scale and measures the same five factors, namely: Past Negative (four items, e.g., “*I think about the bad things that have happened to me in the past”*), Past Positive (three items, e.g., “*I enjoy stories about how things used to be in the good old times”*), Present Hedonistic (three items, e.g., “*I take risks to put excitement in my life”*), Present Fatalistic (three items, e.g., “*My life path is controlled by forces I cannot influence”*) and Future TP (four items, “*I complete projects online by making steady progress”*). Participants indicated their answers on a five-point scale (1 = *very uncharacteristic*; 5 = *very characteristic*).

The short version of **Hypersexual Behavior Inventory** [HBI; (16, 24)] is an eight-item self-report scale assessing hypersexuality. The short version of the scale has a unifactorial structure (e.g., “*Even though I promised myself I would not repeat a sexual behavior, I find myself returning to it over and over* again”). Participants indicated their responses on a five-point Likert scale (1 = *never*; 5 = *very often*).

The short version of **Problematic Pornography Consumption Scale** [PPCS; (77, 78)] was developed to measure the extent of PPU based on Griffiths' six-component addiction model (79). The 6-item scale have a unifactorial structure (e.g., “*I became stressed when something prevented me from watching porn*”). Participants indicated their answers on a seven-point Likert scale (1 = *never*; 7 = *all the time*).

After standard demographic questions (gender, age, relationship status, work status, education level), additional items were asked about participants' number of sexual partners in their lifetime (16-point scale, 1 = *0 partner*, 16 = *more than 50 partners*) and frequency of sexual activities in the past year (10-point scale, 1 = *never*, 10 = *6 or 7 times a week or more*) (77). In the first data collection (Sample 1) frequency of pornography consumption in the last year (11-point scale, 1 = *never*, 11 = *more than 7 times a week)* was also assessed.

### Statistical analysis

IBM SPSS 27 (SPSS Inc., Chicago, IL, USA) (80) was used for data cleaning, and Mplus 8 (81) was used for multivariate analysis. The same statistical analyses were conducted in the two different samples, to examine the robustness of the findings. Normality was assessed by the investigation of skewness and kurtosis values. Reliability was measured by Cronbach's alpha, using Nunnally's (82) guideline concerning its values (≤ 0.7 is acceptable, ≤ 0.8 is good), and McDonald's Omega (83).

Structural equation modeling (SEM) was used to examine the associations between the five domains of TPs, hypersexuality, and PPU. Because of the floor effect in many cases (i.e., hypersexuality and PPU) the items were treated as categorical indicators, thus the Mean- and Variance—Adjusted Weighted Least Square Estimator (WLSMW) was used (84). Following previous studies in sexuality-related topics (85–87) to investigate possible gender differences, multigroup analyses were conducted after the baseline model (Model 1), with gender as a grouping variable (Model 2). In the third model, all paths between the five domains of TPs, hypersexuality, and PPU were constrained to be equal across genders, as well as the correlations between the above-mentioned constructs (Model 3). Goodness of fit was assessed (88, 89) by examining commonly used goodness-of-fit indices (88, 90) the Root-Mean-Square Error of Approximation (RMSEA; ≤ 0.06 for good, ≤ 0.08 for acceptable), the Tucker-Lewis Index (TLI; ≥0.95 for good, ≥0.90 for acceptable) and the Comparative Fit Index (CFI; ≥0.95 for good, ≥0.90 for acceptable) with 90% confident intervals. To compare the two models (constrained and unconstrained models) the changes in chi-square, TLI, CFI and RMSEA indices were examined. Differences between the models were considered significant when significant corrected chi-square differences, significant decrease in TLI and CFI (ΔCFI ≤ 0.010; ΔTLI ≤ 0.010), and significant increases in RMSEA (ΔRMSEA ≤ 0.015) were observed, following previous guidelines (91, 92).

## Results

Descriptive data and reliability indices of the TPs, hypersexuality, PPU and the sexuality-related questions can be seen in Table 1. The correlations between the aforementioned constructs are presented in Table 2.

**Table 1:** Descriptive statistics, reliability indices, and correlations between time perspectives, problematic pornography use, frequency of pornography use, hypersexuality, number of sexual partners, and frequency of having sex in Sample 1 and Sample 2.

	**Sample 1 Observed range**	**Sample 2** **Observed range**	**Sample 1 Skewness (SE)**	**Sample 2** **Skewness (SE)**	**Sample 1 Kurtosis (SE)**	**Sample 2 Kurtosis (SE)**	**Sample 1 M (SD)**	**Sample 2** **M (SD)**	**Sample 1** **α**	**Sample 2** **α**	**Sample 1** **ω**	**Sample 2** **ω**
1. Problematic pornography use	6–42	6–33	1.83 (0.12)	1.52 (0.13)	3.61 (0.23)	1.86 (0.27)	11.39 (6.64)	10.70 (5.52)	0.86	0.78	0.86	0.78
2. Frequency of pornography use^a^	2–11	–	−16 (0.12)	–	−0.93 (0.23)	–	6.67 (2.53)	13.67 (5.32)	–	–	–	–
3. Hypersexuality	7–35	8–37	1.34 (0.11)	1.30 (0.13)	1.80 (0.21)	1.93 (0.26)	12.40 (5.30)	–	0.86	0.81	0.86	0.80
4. Number of partners^b^	1–16	1–16	0.32 (0.10)	0.65 (0.12)	−1.23 (0.21)	−0.80 (0.23)	7.32 (4.61)	6.54 (4.17)	–	–	–	–
5. Frequency of having sex^c^	1–10	1–10	−1.13 (0.11)	−0.94 (0.13)	0.67 (0.21)	0.01 (0.25)	6.83 (2.20)	6.39 (2.32)	–	–	–	–
6. Past negative TP	1–5	1–5	0.05 (0.10)	0.21 (0.12)	−0.90 (0.21)	−0.80 (0.23)	2.91 (0.97)	2.86 (1.00)	0.83	0.83	0.83	0.83
7. Past positive TP	1–5	1–5	−0.21 (0.10)	−0.16 (0.12)	−0.44 (0.21)	−0.49 (0.23)	3.14 (0.79)	3.12 (0.85)	0.62	0.69	0.64	0.70
8. Past hedonistic TP	1–5	1–5	0.03 (0.10)	−0.06 (0.12)	−0.48 (0.21)	−0.36 (0.23)	3.15 (0.81)	3.03 (0.81)	0.69	0.73	0.76	0.79
9. Past fatalistic	1–5	1–5	0.31 (0.10)	0.43 (0.12)	−0.11 (0.21)	−0.02 (0.23)	2.51 (0.82)	2.56 (0.82)	0.68	0.69	0.68	0.70
10. Future TP	1–5	1–5	−0.41 (0.10)	−0.62 (0.12)	0.15 (0.21)	0.66 (0.23)	3.68 (0.61)	3.49 (0.73)	0.60	0.70	0.61	0.71

*SE, Standard Error; M, Mean; SD, Standard Deviation; α, Cronbach's Alpha; ω, McDonals's Omega.*

a *2 = once in the last year; 3 = 1–6 times in the last year; 4 = 7–11 times in the last year; 5 = once in a month; 6 = 2–3 times a month; 7 = once in a week; 8 = 2–3 times in a week; 9 = 4–5 times in a week; 10 = 6–7 times in a week; 11 = more than 7 times in a week.*

b *1 = 0; 2 = 1; 3 = 2; 4 = 3; 5 = 4; 6 = 5; 7 = 6; 8 = 7; 9 = 8; 10 = 9; 11 = 10; 12 = 11–20; 13 = 21–30; 14 = 31–40; 15 = 41–5; 16 = more than 50.*

c *1 = I did not have sex; 2 = once in the last year; 3 = 1–6 times in the last year; 4 = 7–11 times in the last year; 5 = once a month; 6 = 2–3 times a month; 7 = once a week; 8 = 2–3 times a week; 9 = 4–5 times a week; 10 = 6–7 times a week or more*.

**Table 2:** Correlations between time perspectives, problematic pornography use, frequency of pornography use, hypersexuality, number of sexual partners, and frequency of having sexin Sample 1 and Sample 2.

	**1**.	**2**.	**3**.	**4**.	**5**.	**6**.	**7**.	**8**.	**9**.
1. Problematic ponrography use	–	–							
2. Frequency of pornography use^a, b^	0.56**/ –	–	–						
3. Hypersexuality	0.70**/ 0.52**	0.38**/ –	–						
4. Number of partners^c^	0.01/ −0.05	0.08/ –	0.16**/ 0.21**	–					
5. Frequency of having sex^d^	−0.10*/ −0.19**	−0.13**/ –	−0.09*/ −0.03	0.12*/ 0.14**	–				
6. Past negative TP	0.09/ 0.15**	−0.06/ –	0.16*/ 0.30**	0.03/ −0.01	−0.11*/ −0.04	–			
7. Past positive TP	0.11*/ 0.00	−0.08/ –	0.12**/ 0.05	0.02/ −0.02	−0.01/ −0.03	0.01/ 0.06	–		
8. Present Hedonistic TP	0.18**/ 0.24**	0.12*/ –	0.37**/ 0.24**	0.13**/ 0.09	0.08/ −0.03	0.09*/ 0.18**	0.10*/ 0.07	–	
9. Present Fatalistic TP	0.11*/ 0.10	0.02/ –	0.18**/ 0.10	0.05/ −0.04	−0.04/ −0.122*	0.30**/ 0.40**	0.10**/ 0.15**	0.05/ 0.12*	–
10. Future TP	−0.11*/ −0.12**	−0.10*/ –	−0.23**/ −0.12**	−0.05/ −0.06	0.00/ −04	−0.06/ −0.17**	0.13**/ 0.133**	−0.11**/ −0.13**	−0.13**/ −0.16**

TP, time perspective. Pearson correlations that are significant at the p <0.01 level are marked with ^**^, those that are significant at the p <0.05 level are marked with ^*^. The correlation coefficients below the diagonal represent Sample 1, above the diagonal represent Sample 2.

*^a, d^ 1 = none; 2 = once in the last year; 3 = 1–6 times in the last year; 4 = 7–11 times in the last year; 5 = once in a month; 6 = 2–3 times a month; 7 = once a week; 8 = 2–3 times a week; 9 = 4–5 times a week; 10 = 6–7 times a week; 11 = more than 7 times a week.*

*^b^ Frequency of pornography use was not measured in Sample 2.*

*^c^ 1 = 0; 2 = 1; 3 = 2; 4 = 3; 5 = 4; 6 = 5; 7 = 6; 8 = 7; 9 = 8; 10 = 9; 11 = 10; 12 = 11–20; 13 = 21–30; 14 = 31–40; 15 = 41–5; 16 = more than 50 partners.*

*^c^ 1 = I did not have sex; 2 = once in the last year; 3 = 1–6 times in the last year; 4 = 7–11 times in the last year; 5 = once a month; 6 = 2–3 times a month; 7 = once a week; 8 = 2–3 times a week; 9 = 4–5 times a week; 10 = 6–7 times a week or more*.

**Figure 1:**
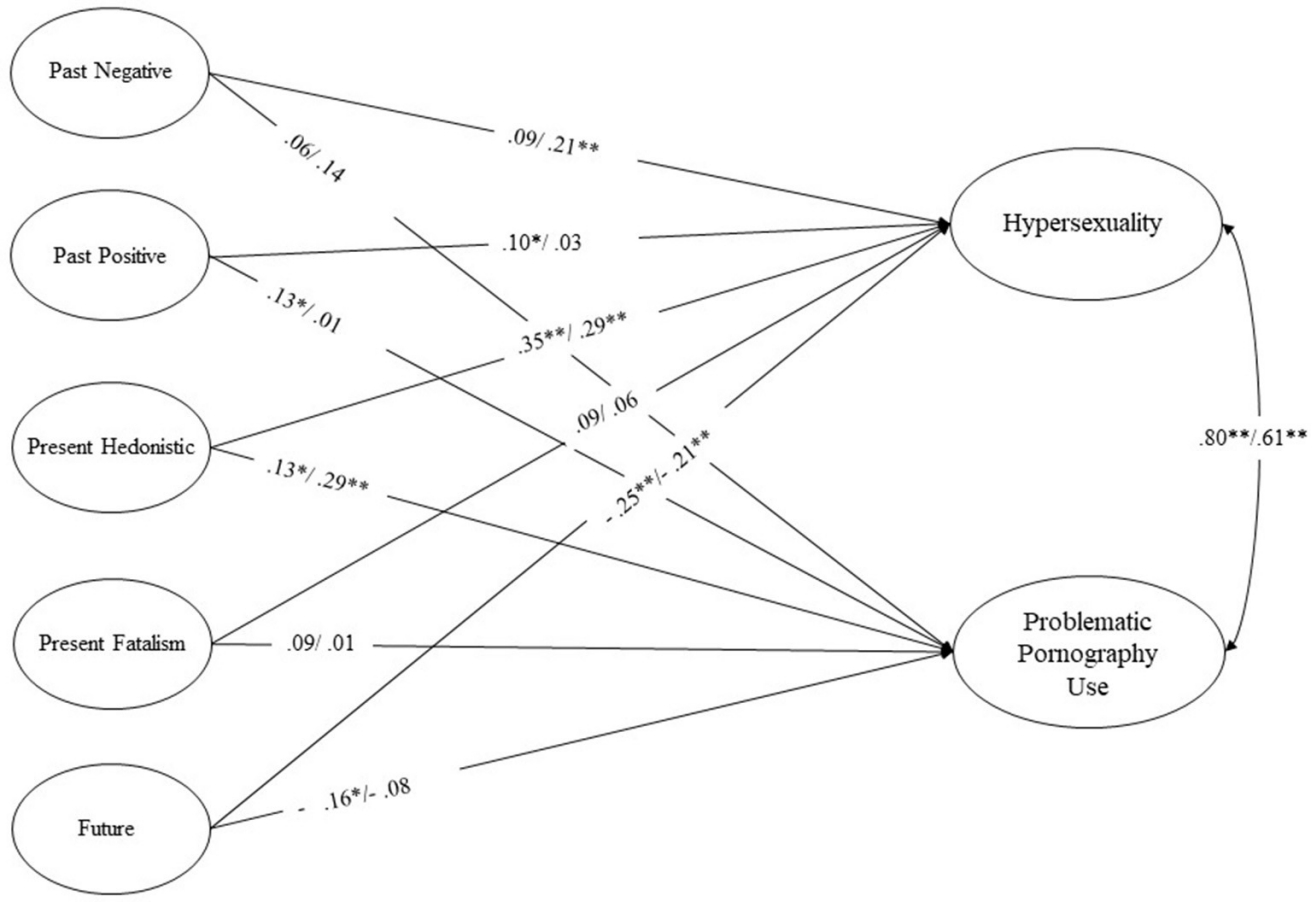
Examining the associations between time perspectives, hypersexuality, and problematic pornography use. All variables presented in ellipses are latent variables. One-headed arrows represent standardized regression weights, and two-headed arrows represent correlations. Numbers on the arrows represent standardized path coefficients (Sample 1 and Sample 2, respectively). Associations that are significant at the *p* < 0.05 level are marked with *, and at the *p* < 0.01 level are marked with **.

Three models were examined assessing the associations between the dimensions of TP, hypersexuality, and PPU. First, an initial model on the total sample (Model 1), then a multigroup analysis with the grouping variable of gender (i.e., men and women) (Model 2), and lastly, a constrained model, where uni-, and bi-directional associations were constrained to be equal across gender-based groups (Model 3), to examine whether the model varies across genders. All estimated models showed acceptable fit to the data, and the changes in model fit indices remained in an acceptable range (Table 3). These results indicate that men and women had similar associations between the different dimensions of TP, hypersexuality and PPU. Therefore, the baseline model (Model 1) was used, following the principle of parsimony. Results of both Sample 1 and Sample 2 are presented by Figure 1.

**Table 3:** Comparison of the associations between time perspectives, hypersexuality, and problematic pornography use among men and women.

**Model**	**WLSMV χ^2^ (df)**	**CFI**	**TLI**	**RMSEA**	**90% CI**	**Comparison**	**ΔCFI**	**ΔTLI**	**ΔRMSEA**	**χ^2^** **Difference test (df)**
**Sample 1**										
M1: Total sample (baseline)	898.85 (413)	0.954	0.948	0.042	0.038–0.046	—	—	—	—	—
M2: Grouping by gender (men vs. women)	1,494.23 (945)	0.945	0.946	0.042	0.038–0.046	M1–M2	−0.009	−0.002	0.000	
M3: Paths constrained to be equal between men and women	1,459.21 (966)	0.950	0.952	0.039	0.035–0.043	M2–M3	0.005	0.006	−0.003	34.675* (21)
**Sample 2**										
M1: Total sample (baseline)	813.70 (413)	0.940	0.933	0.046	0.042–0.051	—	—	—	—	—
M2: Grouping by gender (men vs. women)	1,331.17 (943)	0.938	0.939	0.043	0.037–0.048	M1–M2	−0.002	0.006	−0.003	—
M3: Paths constrained to be equal between men and women	1,378.99 (964)	0.934	0.936	0.044	0.038–0.049	M2–M3	−0.004	−0.003	0.001	52.392* (21)

WLSMV = weighted least squares mean- and variance-adjusted estimator; χ^2^ = Chi-square; df = degrees of freedom; CFI = comparative fit index; TLI = Tucker-Lewis Index; RMSEA = root-mean-square error of approximation; 90% CI = 90% confidence interval of the RMSEA; ΔCFI = change in CFI value compared to the preceding model; ΔTLI = change in the TLI value compared to the preceding model; ΔRMSEA = change in the RMSEA value compared to the preceding model. *p <0.05.

### Time perspective dimensions in relation to hypersexuality

In both samples, Future TP had a moderate and negative association, and Present Hedonistic TP had a moderate and positive association with hypersexuality. Present Fatalistic TP did not have significant associations with either hypersexuality or PPU in any samples. The two remaining TPs showed inconsistent associations with hypersexuality in the two samples. Past Negative TP had a significant, positive, and weak association in Sample 2, but was not significantly related to hypersexuality in Sample 1. Meanwhile, Past Positive TP had positive but weak association with hypersexuality in Sample 1 and showed no significant association with it in Sample 2. The explained variance of hypersexuality was 28.1% in Sample 1, and 27.0% in Sample 2.

### Time perspective dimensions in relation to problematic pornography use

PPU showed similar associations as hypersexuality, except for Future TP, which did not show significant association with PPU in Sample 2, but had a significant, negative and weak association with it in Sample 1. Neither Present Fatalism nor Past Negative TP showed significant associations with PPU in any samples. Past Positive TP had significant, positive but weak association in Sample 1, but not in Sample 2. The explained variance of PPU was 9.8% and 13.7% in Samples 1 and 2, respectively.

## Discussion

The TP theory was associated with different risk-taking behaviors (28, 44–46), substance use disorders (47–50, 73, 74), and with behavioral addictions, such as gambling (93, 121) or online gaming (94). Hypersexuality and PPU has never been examined in relation to the TPs before, but considering the findings in case of other addictions and risk-taking behaviors, similar relationship patterns were hypothesized. According to the results of the present study, Present Hedonistic TP was moderately and positively related to hypersexuality and PPU consistently in both samples, and these associations were the strongest in both samples. Future TP was negatively related to hypersexuality consistently in both samples, and it was also negatively associated with PPU, but only in the first sample. In general, the associations were stronger in relation to hypersexuality. Present Fatalism was unrelated to both problematic sexual behaviors. The two past TPs showed differentiated relationship patterns through the samples. The associations between the TPs and the examined problematic sexual behaviors (i.e., hypersexuality and PPU) were mostly weak. No significant gender differences were observed regarding the associations between TPs and both hypersexuality and PPU. Although the examination of gender-differences in sexuality-related investigations are well-founded (25, 77), these findings support the notion that differences might be smaller than previously presumed in case of out-of-control sexual behaviors (1, 5).

### Time perspective dimensions in relation to hypersexuality

In case of hypersexuality, the hypothesized patterns were identified in both samples. Namely, hypersexuality was negatively associated with the Future, and positively associated with the Present Hedonistic TPs. These results are in line with previous findings regarding the associations between TPs and other risk-taking and problematic behaviors (49, 72–74). These findings suggest that individuals with higher “*carpe-diem*” mentality and lower future goal-orientation may experience higher levels of hypersexuality, as they want to seek pleasure in the present moment and do not strongly consider the potential negative consequences in the future.

In addition, positive associations were observed between the Past Positive TP in Sample 1, and the Past Negative TP in Sample 1 and hypersexuality. Although the former association was weak and not robust, since it was significant only in one sample, it was still unexpected. Partly because none of the previous studies about other addictions had found any similar relations before, and partly because while Past Negative TP tend to correlate negatively with several wellbeing factors (30, 31), the Past Positive TP usually correlates positively with them (33, 95). Zimbardo and Boyd (27) even found that people who score high on the Past Positive scale tend to have less sex, and fewer sexual partners. Furthermore, the past orientations are prone to affect mood and emotions, rather than behavior (27–29).

For the positive association between the Past Negative TP and hypersexuality, one possible explanation might be that hypersexual behaviors can be considered as a potential maladaptive way of coping with traumatic childhood sexual abuse (96–99), leading to lower relationship quality and satisfaction (100). The diagnostic guidelines for Compulsive Sexual Behavioral Disorder (ICD-11; World Health Organization, 2018) include that CSBD in adulthood has been associated with higher rates of childhood traumas, including sexual abuse. The Past Negative TP incorporates items about often thinking, or in the contrary, trying not to think about negative childhood memories, which may relate to traumatic sexual experiences among other negative incidents, and even contains an explicit item about abuse (i.e., “*I've taken my share of abuse and rejection in the past*”). Therefore, the potential association between the Past TPs and hypersexuality warrant further investigation.

### Time perspective dimensions in relation to problematic pornography use

In the case of PPU, the relationship patterns were rather inconsistent across samples, and the explained variance by the TP dimensions was lower than in the case of hypersexuality. In the first sample, the expected associations were identified, namely, PPU had a negative relationship with the Future TP and a positive association with the Present Hedonistic TP. These associations were also in line with previous findings about the TP theory and problematic, out-of-control behaviors (28, 72–74), and could be interpreted similarly as in the case of hypersexuality.

However, in the second sample, the association between the Future TP and PPU was not significant. The inconsistent and small associations with the Future TP across the samples might resemble the variance in the possible negative consequences in the case of PPU. Given that it is mostly a solitary sexual act, the impact of one's out-of-control behavior does not extend to others instantly. Therefore, it might not require having a blindfold regarding the future consequences to the same extent, in contrast with hypersexuality, where the negative consequences can be instant and direct. For example, PPU was associated with relationship and sexual functioning difficulties previously (85, 101), while hypersexuality (in addition to the former consequences) was also associated with legal problems an direct health risks (16, 25). In addition, a positive association could be observed between the Past Positive TP and PPU. This relation to the Past Positive TP was just as unexpected, as it was in case of hypersexuality and require further examination.

In sum, the associations—when they were significant or consistent, like in case of the Present Hedonistic TP—remained weaker in general, than in case of hypersexuality, suggesting that PPU might have less impulsivity-related features, than hypersexuality (61, 64, 102, 103). Furthermore, the explained variances were only the third of what was observed in the case of hypersexuality.

### Comparing hypersexuality and problematic pornography use

It was expected that hypersexuality and PPU would show somewhat similar association patterns with the TPs, as well as many out-of-control behaviors before (28, 72–74). These assumptions were supported in the case of hypersexuality and partly in the case of PPU.

Kafka (22) in his proposed diagnostic criteria for hypersexual disorder considered PPU as a potential manifestation of hypersexuality. In the recent update of the diagnostic criteria of compulsive sexual behavior disorder (12), the use of pornography is mentioned as a possible expression of CSBD. However, recent studies challenged the idea of PPU being a subcategory of hypersexuality (23, 25, 64, 104), suggesting that the two problematic behaviors might have slightly different backgrounds, despite the reported similarities. The present study demonstrates minor differences between the two out-of-control sexual behaviors, suggesting that hypersexuality might more strongly resemble other addictive behaviors (e.g., regarding the impulsive feature of them) than PPU.

### Future studies and limitations

The present study was cross-sectional, limiting causal inferences. The data were not representative to the population (e.g., it excluded people without internet access) and the study was conducted in a WEIRD (i.e., White, Educated, Industrialized, Rich, and Democratic) country (105), limiting the generalizability of findings. The bias of social desirability is also plausible, although Griffiths (106) suggested that people tend to be more honest online than in a face-to face situation, when the subject is as sensitive as sexuality-related questions. Therefore, future studies should consider working with diverse samples regarding gender, sexual orientation, educational level and socioeconomic status, as well as possible cultural differences. Additionally, implementing longitudinal research design is highly recommended, in order to obtain causal associations.

## Conclusions and implications

Results showed that Present Hedonistic and Future TPs might contribute to hypersexuality and PPU as well, and suggest that these out-of-control sexual activities may have similar associations with certain TPs as substance use disorders or behavioral addictions (47–50, 121). It is established in the literature that a balanced TP [the ability to switch between the dominant temporal frames according to the situation's requirements (27, 107)], or the later refined, optimal profile of balanced TP (low Present Fatalistic and Past Negative, moderate Present Hedonistic and Future, and high Past Positive TPs) (108) might contribute to a healthier life. It can affect mental health by its positive associations with life satisfaction, proactive coping subjective wellbeing, happiness, self-determination, positive affect, vitality and self-confidence and by its negative associations with depression and trait-anxiety (11, 109–113). However, a balanced TP can also be related to health-related behaviors, like exercising, eating breakfast, visiting a doctor or dentist on a regular basis and using drugs, alcohol, and tobacco less frequently (73, 114). Thus, using the TP theory as part of an intervention or prevention program [e.g., (115–118)] for hypersexuality (e.g., making the participants aware of their dominant temporal frames, educating them about the balanced TP theory, or adjusting their influential way of thinking about time) might help reduce the levels and potential negative consequences of hypersexuality, given the more flexibly and improvable nature of TP (115) than personality traits or other transdiagnostic features that have been associated with hypersexuality (61, 64, 65, 119–122).

## Data availability statement

The raw data supporting the conclusions of this article will be made available by the authors, without undue reservation.

## Ethics statement

The studies involving human participants were reviewed and approved by 2018/332 and 2016/286. The patients/participants provided their written informed consent to participate in this study.

## Author contributions

MK, GO, ZD, and BB: conception, design, and interpretation of data. MK and BB: analysis of data. MK: drafting the article. GO, ZD, and BB: revising it critically for important intellectual content. All authors: final approval of the version to be published.

## Funding

This study was supported by the Hungarian National Research, Development and Innovation Office (KKP126835; K135629). BB was supported by a postdoctoral fellowship from the SCOUP Team – Sexuality and Couples – Fonds de recherche du Québec, Société et Culture. MK was supported by the New National Excellence Program of the Ministry of Human Capacities (ÚNKP-21-3). GO was supported by a postdoctoral fellowship STARS from Conseil Regional Hauts de France. Dr. Beáta Böthe was supported by the Banting Postdoctoral Fellowship (Social Sciences and Humanities Research Council, SSHRC).

## Conflict of interest

The authors declare that the research was conducted in the absence of any commercial or financial relationships that could be construed as a potential conflict of interest.

## Publisher's note

All claims expressed in this article are solely those of the authors and do not necessarily represent those of their affiliated organizations, or those of the publisher, the editors and the reviewers. Any product that may be evaluated in this article, or claim that may be made by its manufacturer, is not guaranteed or endorsed by the publisher.
