# Supplementary material for: Present hedonism and future time perspectives predicting hypersexuality and problematic pornography use
Source: Front Psychiatry. 2022 Aug 23;13:914919. doi: 10.3389/fpsyt.2022.914919 (PMC9447492; doi:10.3389/fpsyt.2022.914919)
Supplement: Supplementary file 1 [file Table_1.DOCX]

Appendix 1.

Table A1. Descriptive statistics of the examined samples

| Demographics | Sample 1  (N = 554) | Sample 2  (n = 453) |
| --- | --- | --- |
| Gender (man) | 280 (50.54%) | 185 (40.83%) |
| Mean age in years (SD) | 27.36 (9.39) | 27.51 (10.19) |
| Education |  |  |
| Primary school degree or less | 56 (10.1%) | 12 (2.6%) |
| Vocational degree | 46 (8.3%) | 11 (2.4%) |
| High school degree | 257 (46.4%) | 254 (56.1%) |
| Higher education degree (e.g., bachelors, masters, or doctorate) | 195 (35.2%) | 176 (38.9%) |
| Marital status |  |  |
| Single | 175 (31.6%) | 160 (35.3%) |
| In a relationship | 277 (50%) | 205 (45.3%) |
| Engaged | 30 (5.4%) | 15 (3.3%) |
| Married | 72 (13%) | 73 (16.1%) |
| Studying currently | 257 (46.4%) | 274 (60.5%) |
| Working status |  |  |
| Not working | 115 (20.8%) | 132 (29.1%) |
| Having a full-time job | 305 (55.1%) | 169 (37.3%) |
| Having a part-time job | 74 (13.4%) | 83 (18.3%) |
| Working on ad-hoc basis | 60 (10.8%) | 69 (15.2%) |
| Socioeconomic status |  |  |
| Much worse than average | 5 (0.9%) | 3 (0.7%) |
| Little bit worse than average | 31 (5.6%) | 20 (4.4%) |
| Average | 171 (30.9%) | 123 (27.2%) |
| Little bit better than average | 238 (43 %) | 202 (44.6%) |
| Much better than average | 101 (18.2%) | 99 (21.9%) |
| Among the best | 8 (1.4%) | 6 (1.3%) |
| Residence |  |  |
| Capital city | 175 (31.6%) | 247 (54.5%) |
| County town | 277 (50%) | 62 (13.7%) |
| Town | 3 (13%) | 92 (20.3%) |
| Village | 72 (13%) | 52 (11.5%) |

*Note.* A total of 689 (Sample 1) and 785 (Sample 2) participants completed the surveys. As the target population was adults (i.e., individuals being 18 years old or older), 13 and seven underage individuals were excluded from the samples, respectively. Further six and ten people, who identified as neither men nor women were excluded from the study, due to the small sample size of this group, making the analysis underpowered and impossible to conduct. Lastly, 116 (Sample 1) and 315 (Sample 2) participants who had not had any sexual experience before and had not watched any pornographic materials in the past 12 months were also excluded from the study.
